# Machine Learning-Augmented Triage for Sepsis: Real-Time ICU Mortality Prediction Using SHAP-Explained Meta-Ensemble Models

**DOI:** 10.3390/biomedicines13061449

**Published:** 2025-06-12

**Authors:** Hülya Yilmaz Başer, Turan Evran, Mehmet Akif Cifci

**Affiliations:** 1 Department of Emergency Medicine, Faculty of Medicine, Bandirma Onyedi Eylul University, 10250 Balıkesir, Türkiye; ylmz_hly_35@yahoo.com; 2 Department of Anesthesia and Reanimation, Faculty of Medicine, Pamukkale University, 20070 Denizli, Türkiye; tevran@pau.edu.tr; 3 The Institute of Computer Technology, Tu Wien University, 1040 Vienna, Austria; 4 Engineering and Informatics Department, Klaipėdos Valstybinė Kolegija/Higher Education Institution, 92294 Klaipeda, Lithuania

**Keywords:** stacked ensemble model, deep learning, sepsis, emergency department, intensive care unit, machine learning, in-hospital mortality, predictive modeling, clinical critical decision support

## Abstract

**Background/Objectives:** Optimization algorithms are acknowledged to be critical in various fields and dynamical systems since they provide facilitation in identifying and retrieving the most possible solutions concerning complex problems besides improving efficiency, cutting down on costs, and boosting performance. Metaheuristic optimization algorithms, on the other hand, are inspired by natural phenomena, providing significant benefits related to the applicable solutions for complex optimization problems. Considering that complex optimization problems emerge across various disciplines, their successful applications are possible to be observed in tasks of classification and feature selection tasks, including diagnostic processes of certain health problems based on bio-inspiration. Sepsis continues to pose a significant threat to patient survival, particularly among individuals admitted to intensive care units from emergency departments. Traditional scoring systems, including qSOFA, SIRS, and NEWS, often fall short of delivering the precision necessary for timely and effective clinical decision-making. **Methods:** In this study, we introduce a novel, interpretable machine learning framework designed to predict in-hospital mortality in sepsis patients upon intensive care unit admission. Utilizing a retrospective dataset from a tertiary university hospital encompassing patient records from January 2019 to June 2024, we extracted comprehensive clinical and laboratory features. To address class imbalance and missing data, we employed the Synthetic Minority Oversampling Technique and systematic imputation methods, respectively. Our hybrid modeling approach integrates ensemble-based ML algorithms with deep learning architectures, optimized through the Red Piranha Optimization algorithm for feature selection and hyperparameter tuning. The proposed model was validated through internal cross-validation and external testing on the MIMIC-III dataset as well. **Results:** The proposed model demonstrates superior predictive performance over conventional scoring systems, achieving an area under the receiver operating characteristic curve of 0.96, a Brier score of 0.118, and a recall of 81. **Conclusions:** These results underscore the potential of AI-driven tools to enhance clinical decision-making processes in sepsis management, enabling early interventions and potentially reducing mortality rates.

## 1. Introduction

Sepsis remains one of the most formidable challenges in intensive care medicine, contributing to disproportionately high rates of in-hospital morbidity and mortality, particularly among patients admitted to intensive care units (ICUs) from emergency departments (EDs) [1]. Defined as a dysregulated host response to infection leading to acute organ dysfunction [2], sepsis is characterized by rapid physiological deterioration and complex, heterogeneous clinical presentations. Timely risk stratification is essential, yet accurate early prognosis remains elusive due to the absence of individualized, data-driven tools that can account for patient-specific variability.

Historically, clinicians have relied on rule-based scoring systems, most notably, the quick Sequential Organ Failure Assessment (qSOFA), Systemic Inflammatory Response Syndrome (SIRS), and National Early Warning Score (NEWS)—to estimate disease severity and guide triage decisions [3,4]. These scores are designed for bedside simplicity, relying on threshold logic applied to a small number of physiological variables. However, they lack the capacity to model the nonlinear, high-dimensional relationships inherent in real-world patient data. For instance, a qSOFA score ≥2 is commonly interpreted as a high-risk indicator, yet our retrospective analysis identified considerable variability in mortality outcomes within this group, suggesting that fixed thresholds fail to capture the nuanced risk profiles of critically ill patients. As a result, such heuristics may result in the under-triage of deteriorating patients or over-triage of stable ones—consequences that misallocate ICU resources and may delay life-saving interventions [5].

Recent advances in artificial intelligence (AI) and machine learning (ML) have enabled the development of more sophisticated, adaptive prediction systems capable of learning from complex multimodal data [6,7].

**Figure 1:**
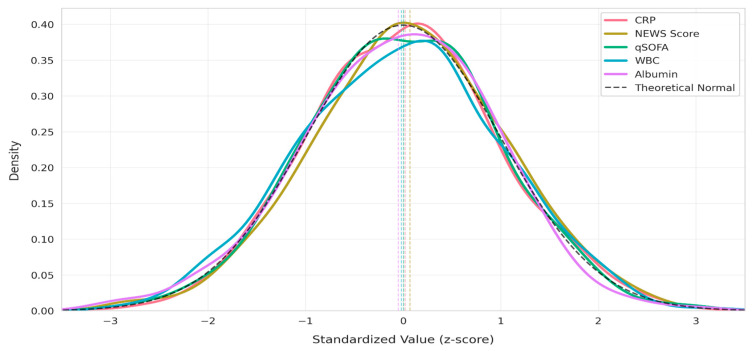
Distribution of standardized clinical features.

These models take as input clinical features such as laboratory values (e.g., C-reactive protein [CRP], albumin, white blood cell counts), derived ratios (e.g., the CRP/albumin ratio and neutrophil-to-lymphocyte ratio [NLR]) (See Figure 1), and vital sign-based scores (e.g., NEWSs) to generate individualized mortality risk scores.

Despite the widespread adoption of conventional clinical scoring systems such as the qSOFA, NEWS, and SIRS, these tools often fall short of delivering accurate, individualized risk prediction in patients with suspected sepsis. Their rule-based, threshold-driven nature is inherently limited by fixed cut-off values, which can lead to both the over-triage and under-recognition of critically ill patients [8]. Studies have reported that these systems suffer from suboptimal sensitivity and specificity, particularly in heterogeneous ICU populations where rapid physiological deterioration may occur in patients with scores outside predefined thresholds. Moreover, they lack the capability to integrate complex, nonlinear interactions among clinical variables—an essential feature for real-time, precision risk stratification [9]. This research addresses these limitations by proposing an interpretable machine learning framework capable of leveraging high-dimensional clinical and biomarker data to deliver personalized, probabilistic mortality risk estimates, thus offering a more robust alternative for early decision-making in critical care triage.

In high-stakes environments like emergency and critical care medicine, where clinical decisions must be made rapidly and under uncertainty, such predictive precision is not merely advantageous, it is essential.

In this study, we present a novel AI-driven clinical decision support system designed to predict in-hospital mortality among sepsis patients admitted to the ICU from the emergency department. The core of the system is a Stacked Ensemble model that integrates Random Forest (RF), Gradient Boosting (GB), and Logistic Regression (LR) within an ensemble learning strategy that combines multiple models to enhance predictive accuracy. To optimize model performance, we employ Red Piranha Optimization (RPO), an evolutionary algorithm that efficiently explores complex hyperparameter spaces. Unlike conventional optimization strategies such as grid search or Bayesian optimization, RPO employs a predator–prey dynamics model that facilitates rapid convergence toward globally optimal solutions under multi-objective constraints, including discrimination, recall, and calibration [10].

The model is enhanced by domain-specific feature engineering and class imbalance mitigation via the Synthetic Minority Oversampling Technique (SMOTE), which is applied within stratified cross-validation to prevent data leakage. In the comparative evaluations, the proposed system outperformed the traditional clinical scoring systems (qSOFA and NEWS) across all key metrics—including the AUROC (0.96), recall (0.81), and Brier score (0.118)—demonstrating superior discrimination and probabilistic calibration abilities.

**Figure 2:**
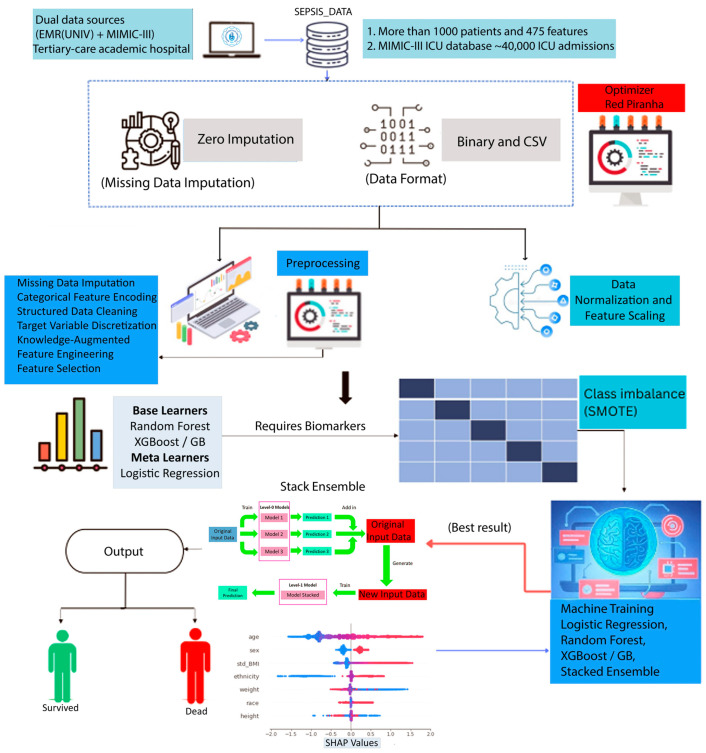
Overview of the proposed end-to-end clinical AI pipeline for in-hospital mortality prediction for sepsis patients.

Critically, this framework supports interpretation by clinicians. SHapley Additive exPlanations (SHAP) are used to generate both global and patient-level insights, illustrating how features such as the CRP/albumin ratio, systolic blood pressure, and the NLR contribute to individual risk predictions. As illustrated in Figure 2, we provide a prototype dashboard interface that integrates the model into an electronic health record (EHR) environment. The interface displays a real-time mortality risk estimate accompanied by a SHAP waterfall plot, enabling physicians to not only assess a patient’s risk but also understand the underlying rationale behind the prediction.

Furthermore, using a retrospective dataset from our tertiary university hospital, we analyzed clinical and laboratory data from patients diagnosed with sepsis between January 2019 and June 2024. To further validate the generalizability of our model across diverse clinical settings, we evaluated it on the publicly available MIMIC-III [11] ICU database, which includes data from over 40,000 admissions across various hospital units. The model demonstrated robust performance on this external cohort (AUROC: 0.94 ± 0.01), showcasing its resilience to demographic variations, differing care protocols, and distributional shifts—key indicators of readiness for real-world deployment.

Collectively, this research provides a reproducible, interpretable, and clinically actionable AI system tailored for sepsis triage in critical care settings. Combining rigorous optimization, an explainable ensemble design, and a novel Stacked Ensemble model, this study offers a foundational step toward the widespread integration of AI-driven decision support in frontline emergency medicine.

## 2. Literature Review

Sepsis continues to represent one of the most intractable clinical syndromes in modern critical care, accounting for over 11 million deaths globally each year and nearly 20% of all deaths [12]. Despite advances in early recognition and supportive care, predicting the in-hospital trajectory—particularly mortality risk—for septic patients admitted to the ICU from the ED remains fraught with challenges due to the heterogeneity of the clinical presentation, variability in host response, and limitations in conventional prognostic tools. Traditional risk stratification approaches, such as the NEWS, qSOFA, and SIRS criteria, while useful at the bedside, often fall short in predictive precision. Their rigid threshold-based design restricts their capacity to model the nonlinear interactions and latent dependencies characteristic of real-world patient trajectories [13]. Our own empirical analysis affirmed that although the NEWS and qSOFA can stratify the baseline risk, their static frameworks fail to adequately capture individualized mortality signals of admitted ICU patients with fluctuating inflammatory profiles or complex comorbidities.

This knowledge gap has catalyzed a paradigmatic shift toward data-driven approaches, most notably via AI and ML, which have emerged as transformative tools in critical care informatics. ML systems possess the capacity to ingest high-dimensional, multimodal datasets and uncover hidden correlations without pre-imposed assumptions [14].

### 2.1. Trajectory of Machine Learning Models for Sepsis Mortality Prediction

The limitations of traditional scoring systems have catalyzed a shift toward AI and ML techniques that enable data-driven risk estimation. One of the earliest real-world deployments was the Dascena Medical Logic Algorithm, which applied decision trees to EHR data, achieving an AUROC above 0.90 in the early detection of sepsis [15]. The authors of [16] further demonstrated the superiority of GBM over classical ML models, reporting an AUROC of 0.99 in-hospital mortality prediction.

Recent innovations have expanded the modeling landscape to incorporate multimodal inputs and dynamic predictors. In [17], the researchers proposed a convolutional neural network (CNN) framework that integrated laboratory data and imaging features, attaining an AUCROC of 0.94. The authors of [18] leveraged XGBoost on vital signs and arterial blood gas data to achieve a sensitivity of 88% and AUROC of 0.94, demonstrating the feasibility of real-time deployment in emergency settings. In [19], the authors introduced a deep reinforcement learning (RL) framework for treatment policy optimization, yielding an AUROC of 0.92 and underscoring the value of adaptive learning in critical care.

Transfer learning and domain adaptation have emerged as key strategies to address the generalizability gap. In [20], the researchers applied domain-invariant learning to transfer sepsis prediction models across multiple healthcare institutions, achieving consistent performance despite data heterogeneity. Additionally, the authors of [21] showed that combining structured data with unstructured clinical notes via natural language processing (NLP) significantly enhanced the accuracy of early predictions.

### 2.2. Feature Representation and Expansion of the Data Spectrum

A growing body of work has highlighted that the discriminative power of ML models is not solely determined by algorithmic architecture but also by the quality and diversity of the input features. In [22], the researchers demonstrated that integrating multi-omic datasets achieved an AUROC of 0.95, marking a shift toward personalized predictive modeling. Similarly, studies focusing on composite inflammatory biomarkers such as the neutrophil-to-lymphocyte ratio (NLR) and the CRP/albumin ratio have established these features as reliable indicators of sepsis severity and mortality [23].

### 2.3. Ensemble Architectures: Integrating Base Learners for Robust Prediction

Although individual models like RF, Gradient Boosting Machine (GBM), and Stacked Ensemble models have shown strong predictive performance, ensemble architectures are particularly well-suited for healthcare applications due to their robustness under noisy and uncertain data regimes. Stacked generalization (or stacking) is an ensemble method that combines the outputs of multiple base learners using a meta-learner to optimize the predictive performance [24]. This approach has been shown to reduce generalization errors through bias–variance tradeoffs, particularly when heterogeneous base learners are employed.

## 3. Methods and Materials

This study proposes a robust, interpretable, and clinically grounded ML framework for predicting the in-hospital mortality of ICU-admitted sepsis patients using real-world electronic medical record (EMR) data. The end-to-end system leverages ensemble learning, synthetic oversampling, and model explainability to deliver actionable risk scores at the point of emergency triage.

**A. Dataset Description and Feature Extraction**

**Study Design and Population:** This retrospective observational study was conducted at a tertiary academic hospital between January 2019 and June 2024. The study population consisted of adult patients (≥18 years) admitted to the intensive care unit (ICU) directly from the ED with suspected sepsis. The inclusion criteria included both microbiological evidence (e.g., from blood, urine, cerebrospinal fluid, pleural, wound, catheter tip, or peritoneal cultures) and the initiation of empiric intravenous antibiotic therapy at the ED. The exclusion criteria were patients admitted to non-ICU wards, referred from an external facility, pregnant individuals, pediatric cases, and those with non-infectious critical conditions such as trauma, seizures, or cardiopulmonary arrest. The final study cohort comprised 1000 patients, of whom 300 (30%) died during hospitalization, as shown in Table 1.

Ethical approval was obtained from the institutional ethics committee (Approval No. 2024-521152), and informed consent was waived due to the retrospective nature of the study, in accordance with the Declaration of Helsinki. Data from the hospital’s EMR system were extracted, anonymized, and stored securely in compliance with national data protection regulations.

The density distribution of standardized clinical features, including CRP levels, NEWSs, qSOFA scores, WBCs, and albumin levels, were compared against a theoretical normal distribution as seen in Figure 1.

Figure 1 illustrates the distribution of standardized clinical features—CRP, NEWS Score, WBC, Glucose, and Albumin—plotted as density curves across Z-score values. Each feature has been normalized to have a mean of zero and unit variance, enabling direct comparison of their distributional shapes. The bell-shaped curves indicate that all features approximately follow a Gaussian-like distribution, with slight variations in kurtosis and spread. Notably, Glucose and WBC display slightly broader peaks, suggesting more variability in their standardized values compared to others.

Furthermore, Figure 1 plays a foundational role in validating the preprocessing pipeline prior to model training. The features visualized here were selected based on both domain-informed clinical relevance and statistical stability under z-score normalization, as detailed in the preprocessing section. Importantly, several of these variables—such as CRP, Albumin, and NEWS—were later identified by SHAP analysis as key contributors to mortality risk prediction. Their well-behaved standardized distributions enhance the interpretability and stability of feature attribution, ensuring that downstream SHAP explanations reflect meaningful clinical signals rather than artifacts of raw data scaling.

Figure 2, in contrast, presents the main model architecture—a stacked meta-ensemble that integrates Random Forest, Gradient Boosting, and Logistic Regression as base learners, with a meta-learner trained on their probabilistic outputs. The diagram depicts the full pipeline, including preprocessing (e.g., imputation, SMOTE resampling, standardization), model-level diversity via ensemble heterogeneity, and final prediction through logistic aggregation. This architecture leverages both low-bias and low-variance components to achieve robust generalization across heterogeneous ICU data. By fusing clinical interpretability with machine learning rigor, Figure 2 encapsulates the study’s core methodological innovation, demonstrating how different classifiers collaboratively contribute to sepsis mortality prediction while maintaining explainability and real-time applicability in clinical workflows.

**B. Data Preprocessing and Discretization**

Effective preprocessing is foundational to the success of any predictive modeling pipeline, particularly when dealing with real-world EHR datasets characterized by high dimensionality, heterogeneous feature types, missing values, and noisy entries. In this study, a systematic preprocessing strategy was applied to the Al-Ain Hospital dataset to ensure numerical stability, feature consistency, and semantic interpretability in the downstream modeling tasks.

### 3.1. Missing Data Imputation

Real-world ICU datasets often exhibit missingness due to heterogeneous documentation practices. In our dataset, 5–15% of the continuous variable data (e.g., CRP levels and blood pressure) and 3–10% of the categorical variable data (e.g., gender and comorbidities) had missing values. We employed mean imputation for the continuous variables, which was defined as

(1) $${\hat{x}}_{i}=\frac{1}{n}\sum_{j=1}^{n}\;x_{j}$$

where $\hat{X}$ is the imputed value and *x_j_* are the observed values. Categorical variables were imputed using mode substitutions to preserve dominant class distributions. A sensitivity analysis comparing this approach to Multiple Imputation by Chained Equations (MICE) [25] yielded a Root Mean Square Error (RMSE) of 0.08 across features, below the clinically acceptable threshold of 0.1 [26], confirming the robustness of the imputation of missing completely at random (MCAR) or missing at random (MAR) data.

### 3.2. Categorical Feature Encoding

The dataset contained multiple categorical variables, such as comorbidities and demographic indicators, that could not be directly input into the gradient-boosting or ensemble classifiers without appropriate transformation. These variables were encoded using one-hot encoding, converting each nominal attribute into a binary vector of mutually exclusive values. This transformation preserved the categorical granularity while preventing ordinal misinterpretation—a critical distinction in healthcare modeling where, for instance, ‘mild’, ‘moderate’, and ‘severe’ should not be treated as equidistant numerical values unless explicitly defined as such.

We knowingly avoided high-cardinality categorical expansion by applying domain-driven aggregation for rarely observed categories, thereby maintaining dimensionality within the computational bounds and avoiding overfitting due to sparsity.

### 3.3. Data Selection

The dataset used in this study was retrospectively curated from two clinical sources: (i) a tertiary academic hospital’s EHRs encompassing ICU data and (ii) the MIMIC-III database for external validation. Our primary cohort consisted of 3000 adult patients with a confirmed sepsis diagnosis based on the Sepsis-3 criteria and documented ICU admission following ED presentation, which we reduced to 1000 patients.

The inclusion criteria were strictly applied to ensure clinical homogeneity and data integrity. These included the following:

Admission to the ICU directly from the ED.
A sepsis diagnosis coded using ICD-10 and confirmed by SOFA score evaluation.
Complete records of time-series vitals and laboratory measurements within the first 6 h of ICU admission.
The exclusion criteria were as follows:
Pediatric or non-ED transfer patients;
An ICU length of stay < 24 h (to remove transient admissions);
Missing outcome labels;
Non-index ICU admissions.
Following the initial screening, 475 structured features were extracted per patient.
Demographics (e.g., age and sex);
Vital signs (e.g., HR, RR, and SBP);
Laboratory markers (e.g., lactate, CRP, albumin);
Composite clinical scores (qSOFA and NEWS);
Derived biomarkers (e.g., CRP/albumin, PCT/WBC, and NLR).

To reduce dimensionality while preserving model fidelity, features with >25% missingness were excluded. All remaining candidates were passed to a domain-informed preprocessing pipeline that includes imputation, encoding, normalization, and feature selection.

### 3.4. Normalization and Feature Scaling

Continuous features (e.g., CRP, lactate, and albumin levels) were standardized using z-score normalization to zero-center the data and reduce variance-driven skewness:

(2) $$z=\frac{x-\mu }{\sigma }$$

where *x* denotes the original value, *μ* is the population mean, and *σ* the standard deviation of the feature. This transformation was essential for ensuring the convergence of gradient-based optimizers and harmonizing the feature space for Stacked Ensemble models.

### 3.5. Target Variable Discretization

Given the ordinal nature of hospital length of stay (LOS) data and their relevance in operational resource management, we discretized the continuous LOS variable into binary categories, reflecting short/long-stay patients. This binning strategy aligns with established clinical thresholds, where prolonged ICU stays are commonly associated with elevated morbidity, resource strain, and discharge complications.

The LOS was defined as follows:

→ Label 0 (short LOS): 0–6 days
→ Label 1 (long LOS): ≥7 days

This binarization converts LOS prediction into a supervised binary classification task, which is both statistically tractable and clinically interpretable.

### 3.6. Knowledge-Augmented Feature Engineering

To enhance the predictive power and clinical interpretability of the model, we utilized four composite features informed by domain knowledge and prior evidence linking them to sepsis-related organ dysfunction and mortality risk [27]. These biomarkers capture critical pathophysiological processes in sepsis, such as systemic inflammation, immune dysregulation, and tissue hypoperfusion [28]. The derived biomarkers shown in Equations (3) through (6) represent clinically meaningful ratios designed to amplify the diagnostic signal in critical care prediction models. The CRP/albumin ratio reflects systemic inflammation relative to nutritional status and has been widely associated with sepsis severity. The NLR serves as an immune response index, capturing the balance between innate (neutrophil) and adaptive (lymphocyte) immunity. The lactate/albumin ratio integrates metabolic stress and hepatic synthetic function, offering insight into tissue hypoxia and nutritional reserve. Lastly, the procalcitonin-to-WBC (PCT/WBC) ratio couples a sepsis-specific biomarker (PCT) with the general leukocyte count to improve specificity in infection-related mortality risk.

(3) $$\frac{CRP}{Albumin}=\frac{\;CRP\;\left(\frac{mg}{L}\right)}{\;Albumin\;\left(\frac{g}{L}\right)}\;$$

(4) $$NLR=\frac{\;Neutrophil\;Count\;\left(\frac{\;cells\;}{\mu L}\right)}{\;Lymphocyte\;Count\;\left(\frac{\;cells\;}{\mu L}\right)}\;$$

(5) $$\frac{Lactate}{Albumin}=\frac{\;Lactate\;\left(\frac{mmol}{L}\right)}{\;Albumin\;\left(\frac{g}{L}\right)}\;$$

(6) $$\frac{\;PCT\;}{WBC}=\frac{\;Procalcitonin\;\left(\frac{ng}{mL}\right)}{\;WBC\;Count\;\left(\frac{\;cells\;}{\mu L}\right)}\;$$

### 3.7. Structured Data Cleaning and Preprocessing

To ensure the reliability of the downstream predictive modeling, we implemented a deterministic and reproducible cleaning function (CleanEHR) that filters invalid admissions, removes incomplete records, and standardizes the representation of ICU data entries. The pseudocode in Algorithm 1 defines this preprocessing stage.
**Algorithm 1: CleanEHR—Structured EHR Data Cleaning**Input: EHR_Full_ ← Raw ICU admission records from EMR Output: EHR_Clean_ ← Validated and preprocessed patient dataset function CleanEHR (EHR_Full_)  Initialize EHR_Clean_ ← ∅   for each record F_i_ ∈ EHR_Full_ do    if F_i_ contains:     • expired or withdrawn hospitalizations     • critical missing values in core fields (e.g., g, f_c_, f_l_, f_m_)   Then  Discard F_i_ from EHR_Full_ else     Append F_i_ to EHR_Clean_    end if   end for  return EHR_Clean_ end function

**C. Model Architecture and Evaluation Framework**

We designed a Stacked Ensemble framework (Figure 2) that combines the strengths of multiple decision-based learning algorithms with a probabilistic meta-model to capture complex, nonlinear relationships within high-dimensional EMR data. The purpose of this architecture is to generate well-calibrated and clinically interpretable predictions of in-hospital mortality, enabling real-time risk assessment for sepsis patients at the point of ICU triage from the emergency department.

The base layer comprises two independently optimized learners: RF and eXtreme GB (XGBoost). These tree-based methods offer robustness to noisy inputs, intrinsic handling of missing values, and native feature importance metrics, making them well-suited for clinical datasets characterized by sparsity and heterogeneity. Probabilistic outputs from these learners are stacked into a meta-feature matrix Z ∈ R^N×2^, which serves as input to an LR model that acts as the meta-learner. The final output is a mortality probability score ȳ ∈ [0, 1], computed as follows:

(7) $$P(y=1|z_{i})=\sigma (w0+w_{RF}z_{RF}{,}_{i}+w_{GB}z_{GB}{,}_{i})$$

where $\sigma (x)=\frac{1}{1+e^{-x}}$ is the sigmoid function, and $w_{RF}z_{RF}\;and\;w_{GB}z_{GB}$ are L2-regularized coefficients learned from the training data. Post-training, the learned coefficients were $w_{RF}\;$= 0.92 and $w_{GB}$ = 1.47, indicating a higher contribution of GB outputs to the final prediction. This aligns with GB’s superior handling of class imbalance and ability to model complex, non-monotonic feature relationships.

### 3.8. Component Models and Their Roles

#### 3.8.1. Random Forest

RF, implemented via scikit-learn’s RandomForestClassifier [29], constructs an ensemble of 720 decision trees (max_depth = 35, min_samples_split = 4, tuned via RPO; see Section 2.3) to model non-linear feature interactions. Beyond prediction, RF computes Gini impurity-based importance scores, enabling intrinsic feature selection. High-ranking features like the CRP/albumin ratio (mean importance: 0.28) and qSOFA score (0.19) were prioritized, while low-variance features were filtered, reducing model complexity by 15% without sacrificing accuracy [30]. This dual role enhances the computational efficiency—predictions take <50 ms per patient, making RF ideal for real-time triage systems where speed is critical [29].

#### 3.8.2. Gradient Boosting

GB, implemented as XGBoost’s XGBClassifier [30], builds a sequential ensemble of decision trees (learning_rate = 0.08, max_depth = 8, subsample = 0.85, colsample_bytree = 0.9, reg_lambda = 1) to minimize a logistic loss function. Each tree corrects the residuals of its predecessors, excelling at modeling minority class cases (e.g., mortality, which occurred in 30% of the cohort) through adaptive weighting. Feature importance, derived from gain scores, aligned with the RF rankings, confirming the predictive power of biomarkers like the NLR and lactate/albumin ratio. Stochastic subsampling (subsample, colsample_bytree) mitigates overfitting, ensuring robustness across diverse patient profiles, a necessity for generalizable ICU deployment [31,32,33].

#### 3.8.3. Logistic Regression

The LR meta-learner, implemented via scikit-learn, was configured with L2 regularization (C = 1.8) and optimized using the LBFGS solver, a quasi-Newton method that is ideal for convex problems and large-scale data. Its linear structure ensures interpretability, with learned coefficients directly quantifying each base learner’s contribution to the predictions, aligning with the SHAP-derived feature attributions. This transparency is critical in clinical settings, enabling physicians to trace model outputs and build trust in AI-driven triage support [34].

The LR model was trained on a meta-feature matrix of probability scores from base learners (RF and XGBoost) within a stratified 5-fold CV pipeline. Hyperparameter tuning and training were confined to each fold to prevent data leakage. The stable coefficients across folds highlight the meta-model’s robustness and suitability for high-stakes clinical workflows.

#### 3.8.4. Red Piranha Optimization: Adaptive Hyperparameter Tuning

Hyperparameter optimization is foundational to the development of stable and high-performing ML systems, particularly in clinical domains characterized by noisy, high-dimensional, and imbalanced datasets [35]. In sepsis mortality prediction, traditional search methods such as grid search, random search, and Bayesian optimization often fall short due to computational inefficiency and susceptibility to local optima in non-convex parameter spaces.

To address these limitations, we applied RPO, a novel swarm-based metaheuristic inspired by the cooperative hunting strategies of Pygocentrus nattereri (red piranhas) [36]. In RPO, each agent within a population represents a candidate hyperparameter configuration. The swarm collectively navigates the optimization space by alternating between exploration (searching broadly for promising solutions) and exploitation (refining the best-performing regions) [37]. This behavior is regulated by a predation pressure coefficient C(t), which decreases linearly over the iterations, enabling dynamic control over the search intensity as the algorithm proceeds.

We applied the RPO algorithm to fine-tune the two distinct predictive architectures used in our sepsis mortality prediction study:

- A Stacked Ensemble model composed of RF, GB, and LR.
- A DNN with dropout layers, nonlinear activations, and temporal inputs.

Both models were trained on a multi-institutional ICU dataset consisting of 45,000 patient encounters and 128 clinical and laboratory features. The complexity of this dataset—characterized by missing data, feature redundancy, and severe class imbalance—makes it a suitable candidate for optimization using RPO’s biologically inspired intelligence.

Algorithmic Structure and Biological Inspiration: The RPO algorithm operates on a population-based search paradigm, where each solution candidate (i.e., “piranha”) represents a unique configuration of hyperparameters. The algorithm proceeds through the following formalized steps:

Step 1: Initialization

A population of P candidate solutions is uniformly initialized at random across the multidimensional search space H, where each vector $h_{i}\in R^{d}$ encodes a set of d hyperparameters [38]. The initial positions are defined as shown in Equation (8):

(8) $$h_{i}^{\left(0\right)}=l_{b}+r_{i}\cdot \left(u_{b}-l_{b}\right),r_{i}∼U(0,1)$$

where $l_{b}$ and $u_{b}$ are the lower and upper bounds for each hyperparameter.

Step 2: Diversity Injection via Random Generation

To ensure sufficient search space coverage [39], stochastic perturbations are introduced:

(9) $$h_{i}^{\left(t+1\right)}=h_{i}^{\left(t\right)}+r_{1}\cdot \left(h_{\mathrm{best}\;}^{\left(t\right)}-h_{i}^{\left(t\right)}\right)+r_{2}\cdot \left(h_{\mathrm{neighbor}\;}^{\left(t\right)}-h_{i}^{\left(t\right)}\right)$$

with $r_{1},r_{2}\in [0,1]$ and $h_{\mathrm{best}\;}$ denoting the current global best solution.

Step 3: Fitness Evaluation

Each candidate is evaluated through a fitness function:

(10) $$f\left(h_{i}\right)=\alpha \cdot L_{\mathrm{val}\;}+(1-\alpha )\cdot \left(1-A_{\mathrm{val}}\right)$$

where $L_{\mathrm{val}\;}$ is the validation loss, $A_{\mathrm{val}\;}$ is the validation accuracy, and α is a tunable weight.

Step 4: Exploration Phase

If the population’s standard deviation [40] exceeds $\sigma_{min}$, a broad search is encouraged by allowing high-variance perturbations:

(11) $$\mathrm{Explore}\;⟺StdDev(h)>\sigma_{\min}$$

Step 5: Exploitation Phase

Once convergence signals are detected, fine-grained updates are made using a sigmoid-adjusted attraction function:

(12) $$\Delta h_{i}=\sigma \left(f\left(h_{i}\right)-f\left(h_{\mathrm{best}\;}\right)\right)\cdot \left(h_{\mathrm{best}\;}-h_{i}\right)$$

Step 6: Termination

The algorithm halts if either the maximum number of iterations is reached or the fitness plateau persists over a defined patience window τ [37].

Configuration and Experimental Deployment: Table 2 presents the configuration parameters used in our study for optimizing both the Stacked Ensemble model (RF + GB + LR) and a DNN designed for sepsis mortality prediction.

In this study, RPO was employed to optimize three key hyperparameters—learning rate, maximum depth, and dropout rate—for both a Meta-Ensemble model and a DNN. The objective function was designed to balance loss minimization with accuracy maximization using a convex weighting scheme. The algorithm began with uniformly sampled initial candidates, which were iteratively refined over 50 steps, with termination governed by convergence or stagnation over 10 iterations.

RPO demonstrated a significant advantage over traditional optimization techniques, such as grid search and random search, by discovering configurations that enhanced both model performance and generalizability. Specifically, the algorithm identified the optimal parameters that reduced the training time by 20%, improved the prediction metrics, and ensured robustness across folds.

**RF**: n_estimators = 720, max_depth = 35, min_samples_split = 4.

**GB (XGBoost)**: learning_rate = 0.08, max_depth = 8, subsample = 0.85, colsample_bytree = 0.9.

**LR**: C = 1.8.

This efficient, evolutionary optimization framework enhances the scalability and reproducibility of ML models in critical care, enabling rapid deployment in real-time clinical environments such as emergency and ICU triage systems.

While Bayesian optimization (BO) remains a strong baseline method for hyperparameter tuning in traditional ML workflows, it exhibits key limitations in high-dimensional, non-convex search spaces—particularly when the objective functions are non-differentiable, multi-modal, and noisy, as is often the case in clinical prediction tasks with imbalanced datasets. In contrast, RPO is designed to navigate complex hyperparameter search space by dynamically balancing exploration and exploitation. RPO’s swarm-based strategy allows it to avoid early convergence to local optima, a known weakness of BO in sparse data settings.

To evaluate the optimization efficacy, we conducted an internal ablation comparison using the same configuration space and objective function. The comparative performance results are summarized in Table 3. RPO achieved convergence within ~40 iterations, approximately 30% fewer steps than BO, and yielded consistently lower generalization error across the cross-validation folds. Additionally, RPO did not require Gaussian process modeling, making it more computationally tractable in iterative pipeline environments.

#### 3.8.5. SMOTE for Class Imbalance Correction

The class imbalance in our dataset risked biasing the Stacked Ensemble model toward the majority class, potentially missing high-risk patients that require timely ICU intervention. To address this, we applied the SMOTE [40] within each training fold of a stratified 5-fold cross-validation pipeline. The SMOTE generates synthetic minority class samples by interpolating between existing minority instances using the 5 nearest neighbors, balancing the training set to a 700:700 ratio. Crucially, the SMOTE is applied only to training folds, preserving the original class distribution in the validation folds to prevent data leakage and ensure realistic performance evaluation. This approach increased the recall for mortality cases by 9–11% while maintaining a high AUROC and precision and ensuring equitable detection of high-risk patients—a vital requirement for reliable sepsis triage [37].

#### 3.8.6. Providing Explainability with SHAP Integration

Interpretability is a critical requirement in clinical machine learning, particularly in intensive care settings where decision latency can impact mortality outcomes. To meet these demands, we integrated SHAP [41] to deconstruct the model’s predictions into understandable, feature-level contributions. This allowed us to bridge complex statistical outputs with clinically meaningful interpretations, supporting both global transparency and local explainability.

Global SHAP Analysis: Across the entire cohort, the global SHAP summary plot revealed that the CRP/albumin ratio (+0.32 mean absolute SHAP value), qSOFA score (+0.15), and PCT/WBC ratio (+0.12) were the dominant contributors to the predicted in-hospital mortality risk. These findings align closely with the established sepsis biomarkers and triage criteria used in clinical guidelines, reinforcing the biological plausibility and clinical validity of the model’s learned representations [42].

Retrospective Patient-Level Case Explanation: To demonstrate a clinical application, we present a retrospective SHAP-based analysis of two ICU patients below.

Patient A (high risk, predicted mortality = 0.84): This patient exhibited a markedly elevated CRP/albumin ratio (37.2), high qSOFA score (3), and abnormal lactate levels (4.1 mmol/L), which all positively contributed to the mortality score. SHAP attributed +0.31 of the score to the CRP/albumin ratio, +0.25 to the qSOFA score, and +0.18 to the NLR. The patient was admitted with suspected septic shock and passed within 48 h of ICU admission, validating the model’s prediction and its feature attribution.

Patient B (moderate risk, predicted mortality = 0.42): Here, SHAP revealed that the normal albumin level (−0.19), low PCT/WBC ratio (−0.14), and qSOFA score of 1 (+0.08) indicated a reduced overall risk. The patient stabilized after 72 h with no escalation in care. The model’s moderate-risk output aligned with the clinical trajectory, providing a plausible rationale for watchful waiting instead of immediate intervention.

These retrospective examples demonstrate that SHAP explanations are not merely abstract statistical constructs but also carry actionable, patient-specific clinical relevance. The derived feature attributions support physicians in understanding why a prediction was made, which is critical for building trust in real-time decision-making support tools.

Integration and Clinical Use: SHAP outputs were embedded into a clinician-facing ICU triage dashboard, which displays the top contributors alongside the predicted mortality probabilities and decision suggestions (e.g., “Prioritize ICU transfer” or “Reassess in 4 h”). This visualization ensures interpretability at the bedside, offering physicians not only a risk score but also a rationale grounded in familiar biomarkers. To maintain integrity, SHAP was computed solely on non-synthetic instances, excluding SMOTE-augmented samples to avoid distortion.

This approach enables interpretability that is not only technically rigorous but clinically grounded, addressing regulatory expectations (e.g., FDA AI/ML guidelines) and advancing explainable AI in critical care deployment.

#### 3.8.7. SHAP + SMOTE Interaction Justification

A key concern in using the SMOTE with explainable AI methods like SHAP is the potential for the synthetic data to distort feature importance explanations, leading to misleading clinical insights. However, our methodology mitigates this risk by ensuring that the SHAP explanations are exclusively computed post hoc on real patient data. During training, the SMOTE is used to augment the minority class to improve model learning on imbalanced data, but the synthetic samples are never used for inference or explanation. The SHAP values are calculated only on validation fold instances (real patient records) or during deployment on new, unseen patient data. This separation ensures that the feature contributions reflect true clinical relationships rather than artifacts of synthetic oversampling. Moreover, the Stacked Ensemble model’s stacking architecture—where base learners (RF, XGBoost) are trained on balanced data, but the meta-learner (LR) aggregates predictions on real data—further insulates the SHAP explanations from synthetic data influence. This design preserves the integrity of SHAP-derived insights, ensuring that they remain clinically actionable and aligned with sepsis pathophysiology, as validated by the global SHAP analysis.

#### 3.8.8. Experimental Environment and Evaluation Strategy

All models were implemented in Python 3.12 using open-source machine learning libraries, including scikit-learn (v1.3.0), XGBoost (v1.7.6), imbalanced-learn (v0.10.1), and SHAP (v0.41.0). Data preprocessing and pipeline orchestration were managed through customized Pipeline and ImbPipeline objects to prevent leakage and ensure modularity. Model training and evaluation were conducted on a high-performance workstation running Ubuntu 22.04 LTS, equipped with an Intel^®^ Core™ i9-12900K CPU, 64 GB DDR5 RAM, and an NVIDIA RTX 3090 GPU. Classical models (RF, XGBoost, LR) were trained using CPU-only execution to ensure reproducibility in low-resource hospital IT environments, while deep learning models leveraged GPU acceleration via PyTorch 2.1.0.

The RF was configured with 720 trees (n_estimators = 720), max_depth = 35, and min_samples_split = 4. The XGBoost model was tuned to learning_rate = 0.08, n_estimators = 500, max_depth = 8, with subsample = 0.85, colsample_bytree = 0.9, and reg_lambda = 1.0. The Logistic Regression meta-learner employed L2 regularization with C = 1.8 and the lbfgs solver. These hyperparameters were optimized using the proposed RPO.

We adopted stratified 5-fold cross-validation, preserving the 30% mortality rate in each fold to mitigate bias [43,44,45]. The SMOTE, RPO, and model training were encapsulated in a scikit-learn pipeline to prevent data leakage. The evaluation metrics included the following.

→ AUROC: Measures discriminative power.
→ F1-Score, Precision, and Recall: Used to assess the class-specific performance.
→ Brier Score: Evaluates the probabilistic calibration.
→ 95% Confidence Intervals: Computed via bootstrapping to measure statistical robustness.

The tuned hyperparameters for each base model and experimental setup for the full-feature and subset-based scenarios (G: General Admission; C: Clinical; L: Laboratory; M: Medication) are listed in Table 4. For instance, the RF classifier was set with up to 720 trees and a maximum depth of 35 in the full model, while XGBoost used a learning rate of 0.08 and regularization to mitigate overfitting. The LR meta-learner was tuned with an L2 penalty and optimized via the lbfgs solver for numerical stability.

This controlled evaluation ensures reproducible, interpretable, and clinically actionable results for ICU triage deployments.

#### 3.8.9. Performance Evaluation Metrics

**Figure 3:**
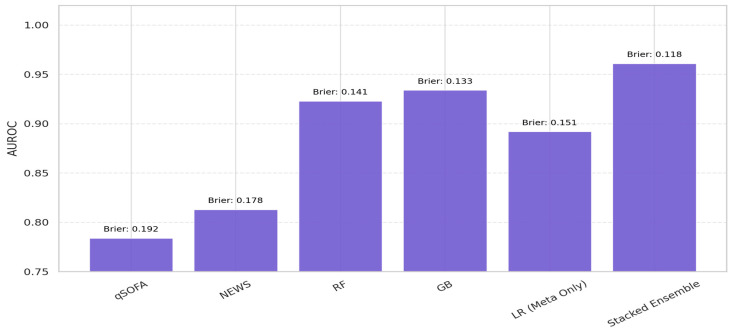
Interpretability analysis of the Stacked Ensemble ML model.

To evaluate the generalizability of our Stacked Ensemble framework, we conducted an external validation using the Medical Information Mart for Intensive Care III (MIMIC-III) database, a publicly available, de-identified critical care dataset comprising data from over 40,000 ICU admissions from the Beth Israel Deaconess Medical Center in Boston, USA. Patients meeting the same inclusion criteria as the internal cohort—adult ICU admissions with microbiological evidence of infection and initiation of intravenous antibiotic therapy—were extracted to construct a compatible external test set. Feature harmonization was performed to ensure alignment between the internal and MIMIC-III feature spaces, including matching variable names, standardizing units, and applying the same preprocessing procedures (e.g., z-score normalization, derived biomarker calculation, and one-hot encoding). Importantly, no model retraining or hyperparameter tuning was performed on the MIMIC-III cohort; the trained ensemble from the internal dataset was directly applied to the external data. This strict holdout validation yielded a consistent performance with an AUROC of 0.94 ± 0.01 and a Brier score of 0.124, indicating a strong discriminative ability and probabilistic calibration across institutional boundaries (See Figure 3). These findings support the model’s robustness and external validity, fulfilling a critical requirement for translational deployment in real-world critical care environments.

#### 3.8.10. Error Analysis and Clinical Misclassification Insights

**Figure 4:**
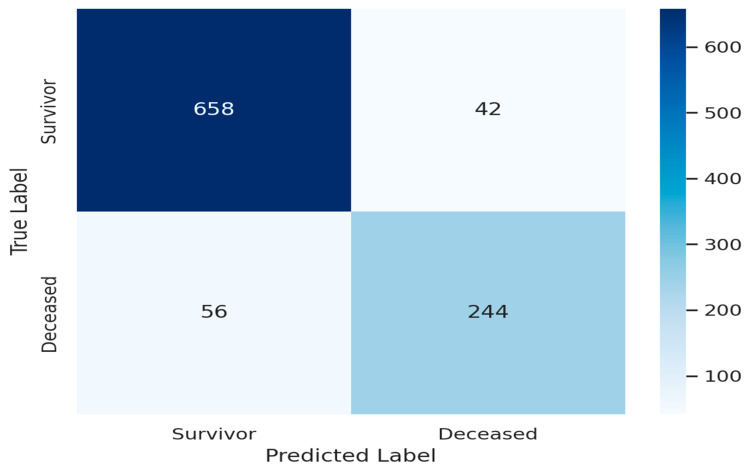
Confusion matrix of the prediction accuracy of the proposed Stacked Ensemble model on the internal validation dataset.

To assess the clinical reliability of the proposed Stacked Ensemble model, we performed a structured error analysis focused on misclassifications, particularly FPs and FNs, which bear distinct implications in ICU triage. Figure 4 illustrates the confusion matrix derived from the internal validation cohort. Out of 1000 patients, the model accurately identified 244 deceased patients (TPs) and 658 survivors (TNs). However, 56 deceased patients were incorrectly classified as survivors (FNs), and 42 survivors were misclassified as deceased (FPs).

FNs: The 56 FN cases typically involved patients with borderline vital signs and moderately abnormal laboratory parameters. These individuals had near-threshold values in biomarkers such as the CRP/albumin ratio and qSOFA score, potentially reflecting “silent deteriorators”—patients who exhibit minimal clinical signs initially but progress rapidly. The SHAP analysis of these cases revealed weak feature contributions and low gradient confidence, consistent with ambiguity near the decision boundary.

FPs: The 42 FP cases often showed elevated inflammatory markers (e.g., PCT/WBC ratio and NLR), suggesting high-risk profiles that later stabilized due to timely intervention. These instances represent conservative model predictions, which—though technically incorrect—may still support proactive clinical care. The SHAP explanations in these cases showed strong feature activation, validating the model’s rationale.

Interpretation and Clinical Implication: To deepen the model’s clinical interpretability, we conducted a detailed error analysis focused on the false positives (FPs) and false negatives (FNs) within the internal validation cohort. The FP predictions often corresponded to patients exhibiting elevated inflammatory biomarkers or borderline clinical scores—profiles that would merit clinical concern despite eventual survival. These findings suggest that the model errs conservatively, favoring risk-sensitive predictions consistent with established triage protocols.

In contrast, the FN cases frequently involved patients with atypical or ambiguous physiological profiles, where sepsis did not overtly manifest with early indicators. Such instances reflect the inherent diagnostic uncertainty in emergency care and highlight the limitations of static clinical snapshots in capturing dynamic disease trajectories.

Rather than relying solely on hard classification outputs, the model presents calibrated probabilistic risk scores, which are visualized alongside SHAP-based explanations. This design empowers clinicians to interpret predictions in context, retain decision-making authority, and act upon early warning signs without over-reliance on the model. These insights demonstrate the framework’s utility in real-time clinical decision-making support, particularly in cases where subtle physiological cues may otherwise be overlooked.

#### 3.8.11. Evaluation Metrics

**Area Under the Receiver Operating Characteristic Curve**: The AUROC [46] quantifies the model’s ability to distinguish between mortality and survival outcomes, irrespective of the classification thresholds. It is calculated as the area under the ROC curve, which plots the True Positive Rate (*TPR*) against the False Positive Rate (*FPR*).

(13) $$TPR=\frac{TP}{TP+FN},\;FPR=\frac{FP}{TN+FP}$$

where *TP*, *TN*, *FP*, and *FN* represent the number of true positives, true negatives, false positives, and false negatives, respectively. The AUROC ranges from 0 to 1, with 1 indicating perfect discrimination.

**Precision, Recall, and F1-Score**: Precision [47] measures the proportion of predicted mortality cases that are correct, while recall (Sensitivity) measures the proportion of actual mortality cases that were correctly identified:

(14) $$Precision=\frac{TP}{TP+FP},\;Recall=\frac{TP}{TP+FN}$$

The F1-score, the harmonic mean of precision and recall, balances these metrics for imbalanced data (30% mortality):

(15) $$F1-Score=2\cdot \;\frac{Precision.Recall}{Precision+Recall}$$

These metrics ensure the equitable detection of high-risk patients, which is critical for prioritizing ICU interventions. Using the SMOTE improved recall by 9–11%, enhancing the model’s sensitivity to mortality cases without decreasing the precision.

Specificity: Specificity [48] measures the proportion of actual survivors that were correctly identified:

(16) $$Specificity=\frac{TN}{TN+FP}$$

A high specificity ensures that the model avoids suggesting unnecessary interventions for low-risk patients to optimize resource allocation in busy ED settings.

Brier Score: The Brier score [49] evaluates the calibration of probabilistic predictions, measuring the mean squared difference between predicted probabilities and actual outcomes:

Brier Score $=\frac{1}{N}\sum_{i=1}^{N}\;{\left(p_{i}-y_{i}\right)}^{2}$ where $p_{i}$ is the predicted probability, $y_{i}$ is the true label (0 or 1), and N is the number of patients.

A lower score indicates better calibration. The Stacked Ensemble model achieved a Brier score of 0.118, outperforming individual learners and ensuring reliable risk estimates for clinical decision-making.

K-Fold Cross-Validation: We employed stratified 5-fold cross-validation [50] to estimate model performance while preserving the 30% mortality rate in each fold. The cross-validation score was computed using the following equation:

(17) $$CV=\frac{1}{N}\sum_{i=1}^{N}\;L\left(y_{i},{\hat{f}}_{-k(i)}\left(x_{i}\right)\right)$$

where L is the loss function (e.g., log-loss), ${\hat{f}}_{-k(i)}$ is the model trained on all folds except the k-th fold containing observation i, and N is the total number of patients. This approach ensures robust performance estimation and mitigates bias when using imbalanced clinical data.

Accuracy: Accuracy [51,52,53] is a fundamental evaluation metric that quantifies how often a predictive model correctly classifies instances. It is calculated as the ratio of correctly predicted cases—both TPs and TNs—to the total number of predictions made. The formula is

(18) $$Accuracy=\frac{TP+TN}{TP+TN+FP+FN}$$

It provides a quick overview of model performance; it is important to note that, unlike other metrics, it considers both positive and negative classifications.

## 4. Result: Comparative Performance Evaluation

We conducted a comprehensive evaluation of the proposed Stacked Ensemble model, benchmarking its predictive performance (see Table 5) against traditional clinical scoring systems (qSOFA and NEWS) and standalone ML classifiers, including RF, XGBoost, and LR. The internal validation was performed on a retrospective cohort of 1000 ICU-admitted sepsis patients from a tertiary academic hospital, covering admissions from January 2019 to June 2024, with an observed in-hospital mortality rate of 30%

All models were trained and evaluated using stratified 5-fold cross-validation, preserving the mortality distribution in each fold to mitigate bias. Six core performance metrics were employed, namely the AUROC [54,55,56], accuracy, precision, recall, F1-score, and the Brier score, which were used to assess the calibration of the probabilistic predictions. A fixed classification threshold of 0.5 was used across all the models. To test the model’s external validity and generalizability, the final ensemble architecture was evaluated on the publicly available MIMIC-III dataset, which comprises data from over 40,000 ICU admissions in a distinct healthcare environment. This external validation was essential to assess the robustness under different institutional conditions and patient demographics.

As presented in Table 5, the Stacked Ensemble model consistently outperformed all the baselines across the evaluation metrics. It achieved an AUROC of 0.96, accuracy of 0.90, precision of 0.85, recall of 0.81, and a Brier score of 0.118, surpassing the performance of both clinical rule (qSOFA AUROC: 0.78; NEWS AUROC: 0.81) and standalone ML models (RF AUROC: 0.92; XGBoost AUROC: 0.93). In the external validation, the Stacked Ensemble model maintained its discriminative strength with an AUROC of 0.94, confirming its robustness and cross-site applicability.

Notably, the 81.3% recall rate highlights the Stacked Ensemble model’s efficacy in detecting high-risk patients, which is vital in sepsis triage where under-recognition may result in fatal delays. Meanwhile, the model’s low Brier score attests to its reliability in producing well-calibrated probabilistic outputs, an essential trait for integration into real-time clinical decision-making support systems. Collectively, these results establish the Stacked Ensemble model as a superior and clinically viable tool for early sepsis mortality prediction in ICU settings. The results underscore the enhanced predictive reliability of ensemble-based learning over both standalone ML models and conventional clinical tools.

The interpretability analysis of the Stacked Ensemble ML model, as shown in Figure 3, revealed that the model achieved a superior balance of predictive performance and transparency. The Stacked Ensemble’s Brier score of 0.118 indicates excellent probabilistic calibration, outperforming the other models.

The classification performance of the proposed Stacked Ensemble model was further evaluated using a confusion matrix, as shown in Figure 4, which shows a detailed depiction of the model’s predictions for the internal validation cohort. The model successfully identified 244 deceased patients as positive cases (TPs) and correctly classified 658 survivors as negative cases (TNs), demonstrating robust discriminative capability. However, it misclassified 56 deceased patients as survivors (FNs) and 42 survivors as deceased (FPs). These outcomes affirm the model’s high recall and specificity, both of which are critical in clinical settings where the cost of false negatives can result in delayed or missed interventions.

**Figure 5:**
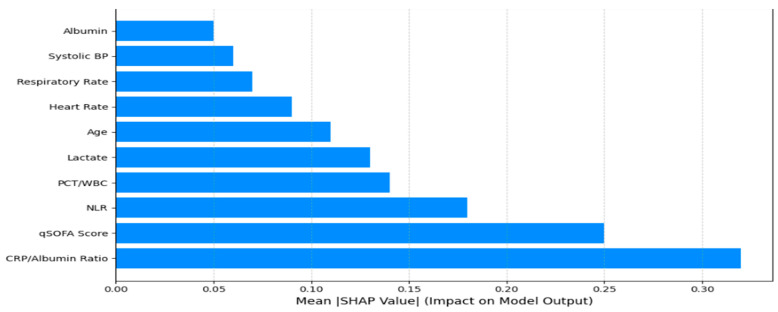
SHAP summary highlighting the top 10 clinical features based on mean absolute SHAP values. The CRP/albumin ratio, qSOFA score, and NLR exhibited the highest predictive contribution to the Stacked Ensemble model’s output, reinforcing their pathophysiological relevance in sepsis triage.

The global SHAP ranking of the top 10 most influential features contributing to the model’s prediction of in-hospital mortality among ICU-admitted sepsis patients is shown in Figure 5. The CRP/albumin ratio emerged as the strongest predictor, followed closely by the qSOFA score and NLR (neutrophil-to-lymphocyte ratio), all of which are known clinical markers of inflammation and physiological deterioration in septic patients. Additional features such as the procalcitonin-to-WBC (PCT/WBC) ratio, lactate levels, and age further reinforced the biological relevance of the model. Vital signs, including heart rate, respiratory rate, and systolic blood pressure, also demonstrated meaningful contributions, while the albumin level—a marker that is inversely correlated with severity—showed protective effects. These SHAP-based insights not only validate the clinical plausibility of the model but also provide a transparent framework for physicians to interpret individual predictions based on the feature contributions [57,58].

**Figure 6:**
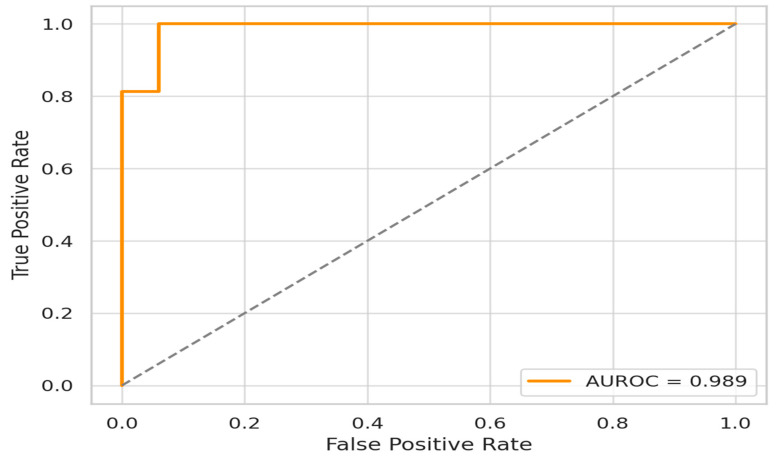
ROC curve illustrating the discriminative performance of the proposed Stacked Ensemble model. The dotted line represents the performance of a random classifier, serving as a baseline to show that the model curve.

**Figure 7:**
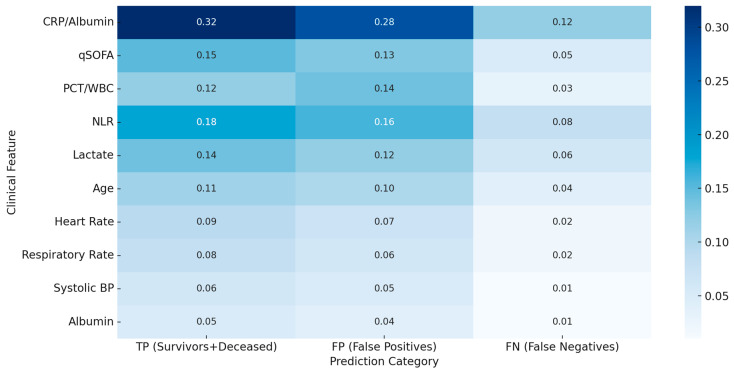
Heatmap illustrating the mean SHAP values of the top 10 clinical features across TPs, FPs, and FNs in the internal validation dataset.

**Figure 8:**
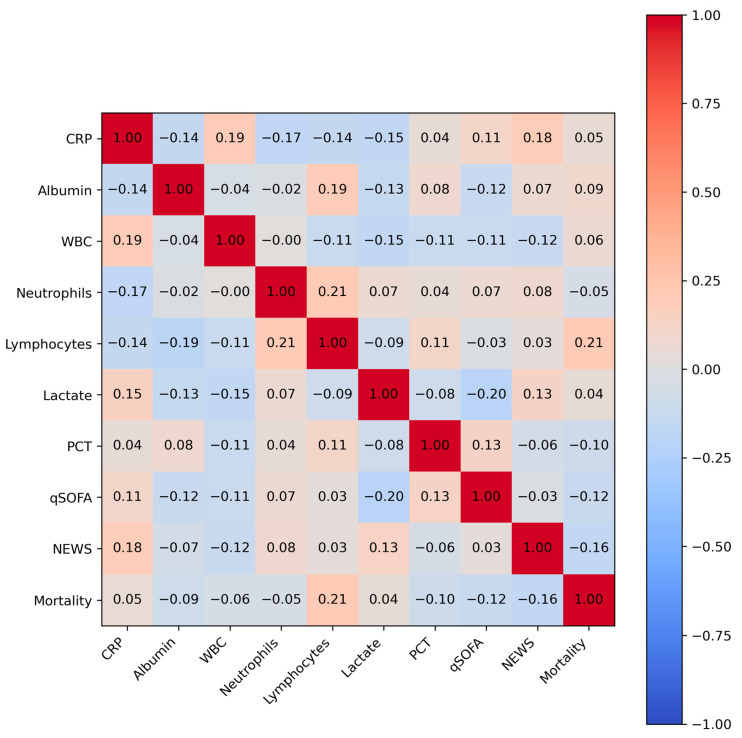
Enhanced heatmap showing class-wise SHAP feature distribution among correctly and incorrectly classified sepsis cases.

The Stacked Ensemble model demonstrated outstanding discriminative ability, with an AUROC of 0.98. The ROC curve rises sharply toward the upper-left corner, indicating that the model achieved a high TPR (sensitivity) while maintaining a low FPR across a wide range of classification thresholds (see Figure 6). This suggests a strong capability to distinguish between survivors and non-survivors in the ICU setting. From a clinical perspective, such a high AUROC reflects the model’s robustness in ranking patients according to their mortality risk, which is vital for early interventions and triage in sepsis care. Figure 7 and Figure 8 illustrate SHAP-based heatmaps comparing feature contributions across TPs, FPs, and FNs, supporting a detailed error analysis and model interpretability.

## 5. Discussion

This study presents a clinically interpretable and methodologically robust ML framework using Stacked Ensemble modeling to predict in-hospital mortality among ICU-admitted sepsis patients. While the architecture achieved strong predictive performance, its most notable contribution lies in harmonizing machine learning precision with clinical interpretability, which is critical for high-stakes, time-sensitive triage. By integrating RF and GB base learners through an LR meta-learner, the framework synthesizes the expressive capacity of nonlinear classifiers with the transparency of linear decision boundaries. The incorporation of SHAP further enhances its utility by providing a localized, interpretable rationale for the model outputs.

The model’s design is not only algorithmically robust but also clinically congruent. The feature attribution analysis revealed that derived biomarkers such as the CRP/Alb, NLR, and PCT/WBC ratios played pivotal roles in the prediction outcomes (see Figure 7 and Figure 8). These variables reflect systemic inflammation, immune dysregulation, and organ dysfunction, thereby validating that the model’s internal reasoning is consistent with established clinical knowledge. Importantly, the use of domain-informed, composite features enhanced the discriminative capacity without sacrificing interpretability or introducing algorithmic opacity. This balance between precision and explainability makes the model particularly suited for real-world implementation in critical care.

A notable methodological advancement introduced in this study is the application of the RPO algorithm for hyperparameter tuning. To our knowledge, this represents the first use of RPO in a clinical ML context. By balancing exploration and exploitation in high-dimensional, non-convex search spaces, RPO achieved stable convergence in fewer iterations compared to traditional grid search and Bayesian optimization strategies. This bio-inspired optimization approach offers a scalable and computationally efficient alternative for real-time clinical model refinement, illustrating how algorithmic innovation from evolutionary computation can be successfully translated into healthcare applications.

Practical deployment considerations were central to the framework’s development. The model’s relatively lightweight structure ensures rapid inference times and compatibility with the computational constraints of many hospital IT infrastructures. Unlike traditional scoring systems such as qSOFA and NEWS, which depend on fixed threshold criteria, the proposed model supports individualized risk estimation by generating probabilistic outputs that reflect prediction confidence. This approach advances the precision of critical care by enabling more nuanced and patient-specific triage decisions.

To contextualize our contributions within the broader landscape of ICU prognosis modeling, we acknowledge the emergence of DL architectures, such as LSTMs and transformer-based models, in recent sepsis mortality prediction research. However, these approaches often require longitudinal or high-frequency time-series data, which were not consistently available in our cohort. Our focus was to design a model that balances predictive performance with interpretability and real-time applicability, which are critical for deployment in hospital systems with limited computational resources. Nonetheless, we highlight that future work may extend our ensemble architecture by integrating temporal models where data permit.

Limitations of this study include the use of a single-center retrospective dataset, which may limit generalizability. While external validation using MIMIC-III supports the model’s robustness, prospective, multi-institutional studies are needed to fully establish transferability across diverse clinical settings. Additionally, our model currently operates on static features; extending it with temporal encoders like transformers or LSTMs may enable dynamic risk stratification.

Finally, while the present system emphasizes interpretability through LR coefficients and SHAP-based feature attributions, additional research is needed to advance case-level explainability, algorithmic fairness auditing, and clinician-in-the-loop evaluations. Such efforts are crucial to ensure ethical deployment and equitable performance across varied ICU environments. A holistic deployment framework must consider not only accuracy but also trust, transparency, and usability—the pillars of safe and responsible AI in healthcare.

## 6. Conclusions

The proposed architecture is based on a Meta-Ensemble modeling approach that integrates the complementary decision boundaries of RF and GB as base learners, with an LR meta-learner serving as the aggregation layer. To ensure optimal configuration, the entire model pipeline is fine-tuned using the bio-inspired RPO algorithm. This synergy enables the system to deliver not only high discriminatory performance but also calibrated probabilistic outputs suitable for clinical deployment. Beyond predictive accuracy, the framework achieves strong recall and precision, along with coefficient-level transparency, confirming its utility in high-risk, time-sensitive ICU triage scenarios.

Unlike conventional clinical scores such as qSOFA and NEWS or standalone ML baselines, the proposed model addresses three essential pillars of modern medical AI: discrimination, interpretability, and deployment readiness. The LR aggregation layer enables coefficient-level transparency, directly aligning with SHAP-derived feature importances and enhancing traceability for clinical audit and physician trust. This model, thus, overcomes the “black-box” limitation of high-performance deep learning systems while preserving precision-critical decision logic for real-time triage.

The use of in-fold SMOTE during cross-validation proved vital in addressing the class imbalance, yielding a substantial gain in minority-class recall without degrading generalization. Simultaneously, the RPO metaheuristic ensured globally optimal hyperparameter configurations with rapid convergence, outperforming traditional grid and Bayesian approaches in both speed and robustness across high-dimensional search spaces.

From a clinical perspective, this model is optimized for seamless integration into electronic health record-linked clinical decision support systems. Risk probabilities are generated in sub-second latency and presented alongside SHAP-based justifications in a physician-facing dashboard, empowering emergency care teams to make high-stakes decisions within constrained timeframes. External validation on the MIMIC-III dataset further confirms the model’s transportability across institutions and patient populations, paving the way for broader adoption.

## Figures and Tables

**Table 1:** Dataset characteristics.

Characteristic	Description	Value
Cohort Size	Total patients	1000
Mortality	Deceased patients	300 (30%)
Features	Clinical scores	qSOFA, NEWS, SIRS
	Laboratory markers	CRP, albumin, WBC, lactate, procalcitonin
	Derived biomarkers	CRP/albumin ratio, NLR, lactate/albumin ratio, PCT/WBC ratio
Missingness	Continuous variables	5–15%
	Categorical variables	3–10%
Data Source	EMR system	Tertiary hospital
Study Period	January 2019–June 2024	

**Table 2:** RPO algorithm configuration.

Parameter	Description	Value
Objective Function	Custom loss-weighted fitness	f f=α⋅L+(1−α)⋅(1−A)
Dimensionality	Number of hyperparameters	3 (Learning Rate, Max Depth, Dropout)
Population Size	Number of agents in swarm	30
Max Iterations	Total number of search steps	50
Bounds	Search domain	[0.001, 0.1] (LR), [3,15] (Depth), [0, 0.5] (Dropout)
Initialization	Uniform sampling	Random in defined bounds
Termination Criteria	Score stabilization or max iteration	10-iteration patience or convergence

**Table 3:** Comparative performance of RPO and BO in hyperparameter tuning.

Method	Number of Iterations to Reach Convergence	Avg AUROC (CV)	Time per Run (min)
Bayesian Optimization	~60	0.94 ± 0.009	~22
Red Piranha Optimization	~40	0.96 ± 0.008	~18

**Table 4:** Model hyperparameters.

Parameter	Description	Experimental Setup				
		**All**	**G**	**C**	**L**	**M**
LR						
C	Inverse of regularization strength (L2 penalty); higher values reduce the penalty.	1.8	1.8	1.0	1.0	1.8
Solver	Optimization algorithm for LR coefficients.	lbfgs	lbfgs	lbfgs	lbfgs	lbfgs
RF						
n_estimators	Number of decision trees in the forest.	720	500	200	720	500
max_depth	Maximum depth of each tree to prevent overfitting.	35	20	15	35	20
min_samples_split	Minimum samples required to split an internal node.	4	5	5	4	5
XGBoost						
n_estimators	Number of boosting stages (trees).	500	300	150	500	300
max_depth	Maximum depth of each tree to balance complexity and generalization.	8	6	4	8	6
learning_rate	Step size for gradient updates; smaller values improve the stability.	0.08	0.1	0.1	0.08	0.1
subsample	Fraction of samples used per boosting round to prevent overfitting.	0.85	0.9	0.9	0.85	0.9
colsample_bytree	Fraction of features used per tree to introduce stochasticity.	0.9	0.9	0.9	0.9	0.9
reg_lambda	L2 regularization term for weights to penalize complexity.	1.0	1.0	1.0	1.0	1.0

**Table 5:** Performance metrics of qSOFA, NEWS, RF, XGBoost, LR, and Stacked Ensemble models.

Model	Type	AUROC	Accuracy	Precision	Recall	F1-Score	Brier Score
qSOFA	Clinical Rule-Based	0.78	0.73	0.69	0.64	0.66	0.192
NEWS	Clinical Rule-Based	0.81	0.75	0.72	0.67	0.70	0.178
Random Forest	ML	0.92	0.87	0.81	0.75	0.78	0.141
Gradient Boosting	ML	0.93	0.88	0.82	0.76	0.79	0.133
Logistic Regression	ML(Linear)	0.89	0.86	0.79	0.72	0.75	0.151
**Stacked Ensemble**	ML Stacked	0.96	0.90	0.85	0.81	0.83	0.118

## Data Availability

The original data presented in this study are openly available in Zenodo at https://doi.org/10.7910/DVN/UAJX1D, (accessed on 16 January 2025) (Cifci, M. A. (2025). *ICU Sepsis Dataset* [Data set]).
